# Magnetically modulated critical current densities of Co/Nb hybrid

**DOI:** 10.1038/srep18601

**Published:** 2015-12-18

**Authors:** Zhigang Li, Weike Wang, Li Zhang, Zhaorong Yang, Mingliang Tian, Yuheng Zhang

**Affiliations:** 1 High magnetic field laboratory, Chinese Academy of Sciences, Hefei 230031, P. R. China; 2 Department of Physics & Electronic Engineering, Taizhou University, Taizhou 318000, China; 3 Key Laboratory of Materials Physics, Institute of Solid State Physics, Chinese Academy of Sciences, Hefei 230031, China; 4 Collaborative Innovation Center of Advanced Microstructures, Nanjing University, Nanjing 210093, China

## Abstract

By tuning morphology and size of magnetic subsystem, ferromagnet-superconductor (F/S) hybrid system provides an effective way to modulate superconductivity due to the interaction between superconducting and magnetic-order parameters at the mesoscopic length scale. In this work, we report on investigations of critical current density in a large-area Co/Nb hybrid via facile colloidal lithography. Here, Co hexagon shell array as a magnetic template build on Nb film to modulate the critical current density. A novel superconducting transition has been observed in I-V curve with two metastable transition states: double-transition and binary-oscillation-transition states. Importantly, such unusual behavior can be adjusted by temperature, magnetic field and contact area of F/S. Such hybrid film has important implications for understanding the role of magnetic subsystem modulating superconductivity, as well as applied to low-energy electronic devices such as superconducting current fault limiters.

Because many physical properties have fundamental length scales of a few to a few hundred nanometers, such as superconducting coherence length ξ and single magnetic domain critical dimension, compositional control at such length scales provides tremendous possibilities for achieving new combinations of those properties^1^. Thus, hybrid nanostructures provided a versatile way to develop novel properties via the coupling between components interface with different properties^2^, especially magnetism and super-conductivity^3^. During the past several years, intensive research efforts have been focused on hybrid F/S systems, and they aimed at modulating superconductivity with magnetic subsystem^4^,^5^,^6^,^7^,^8^,^9^,^10^, exploration unusual physical phenomena^11^,^12^, and potential application in electromagnetic devices^13^,^14^,^15^,^16^.

Critical current density is the crucial property for the application of superconductor devices^17^. Recently, some researches about F/S with periodic arrays have revealed that the interaction between stray field and magnetic flux in magnetic substructure can pin the vortex in superconductor, thereby improve critical current density^18^,^19^,^20^,^21^,^22^,^23^,^24^,^25^,^26^. Those reports provided a promising method to tune critical current density by a period magnetic template in F/S. Besides, up to date, most of F/S hybrids are based on lithographic techniques^25^,^26^,^27^, or superconductor grown on a ferromagnetic monocrystalline substrate, like BaFe_12_O_19_^28^,^29^. Both of them are not economical to fabricate F/S hybrids. Hence, a low-cost, large scale and high ordered hybrid F/S with adjusting critical current density is highly desired.

Colloidal lithography, a low-cost, facile method based on monolayer or multilayer polystyrene spheres (PS) colloidal crystals, has been proven to be an effective tool for fabricating diversiform periodic patterns with large areas^30^,^31^,^32^,^33^,^34^,^35^. In this paper, we present investigations on transport properties of Co/Nb hybrids. Here, Nb film with a thickness of 150 nm was grown on silicon substrate through magnetron sputtering. Co hexagon shells with a diameter of 1 μm and thickness of 70 nm were arranged on the top of Nb film via colloidal lithography. I-V curves reveal that the magnetic template will modulate critical current densities in hybrid. It was detected a novel superconducting transition with two resistive transition states from superconducting state (SS) to normal state (NS): one transition has two critical current densities, namely, the resistance change from SS to NS twice according to the value of current densities, we term the double-transition; another transition is the critical current density existing within a certain range not a fixed value, namely, the resistance oscillated between SS and NS in a certain range, we term the binary-oscillation-transition. Further study shows that those unusual behaviors could be adjusted by temperature, magnetic field and contact area of F/S.

## Results

**Figure 1:**
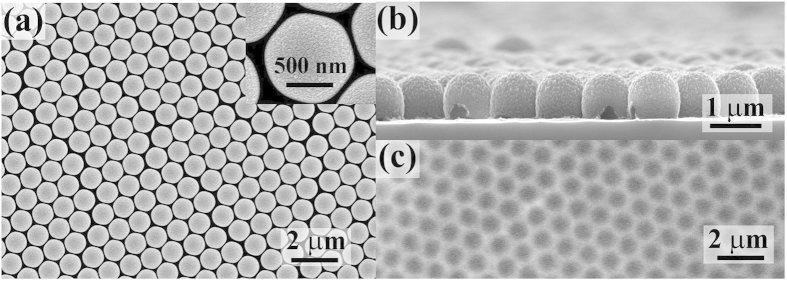
FE-SEM image of Co/Nb hybrid film based on 1 μm PSs template. (**a**) Top view, the inset is local magnification, (**b**) side view, (**c**) the interface of Co shells and Nb film after removing Co shells by ultrasonic cleaning.

Figure 1 presents field-emission scanning electron microscopy (FE-SEM) images of typical Co/Nb hybrid based on a large area (~80 m^2^) high order colloidal template, see supplementary Fig. S1. Figure 1(a) shows the top view of Co shell arrays, which exhibits hexagon arrangement. The inset in Fig. 1(a) is local magnification. Figure 1(b) shows the side view of the hybrid. It can be observed that the Co shell is hollow, and the bottom of shells is connected together. Figure 1(c) shows a honeycomb interface after removing the shell of Co by ultrasonic cleaning. Here, the frame of honeycomb is Co due to physical vapor go through the gaps of PSs and deposition on the bottom of PSs during Co magnetron sputtering. Detailed formation mechanism can be found elsewhere^36^,^37^,^38^,^39^.

**Figure 2:**
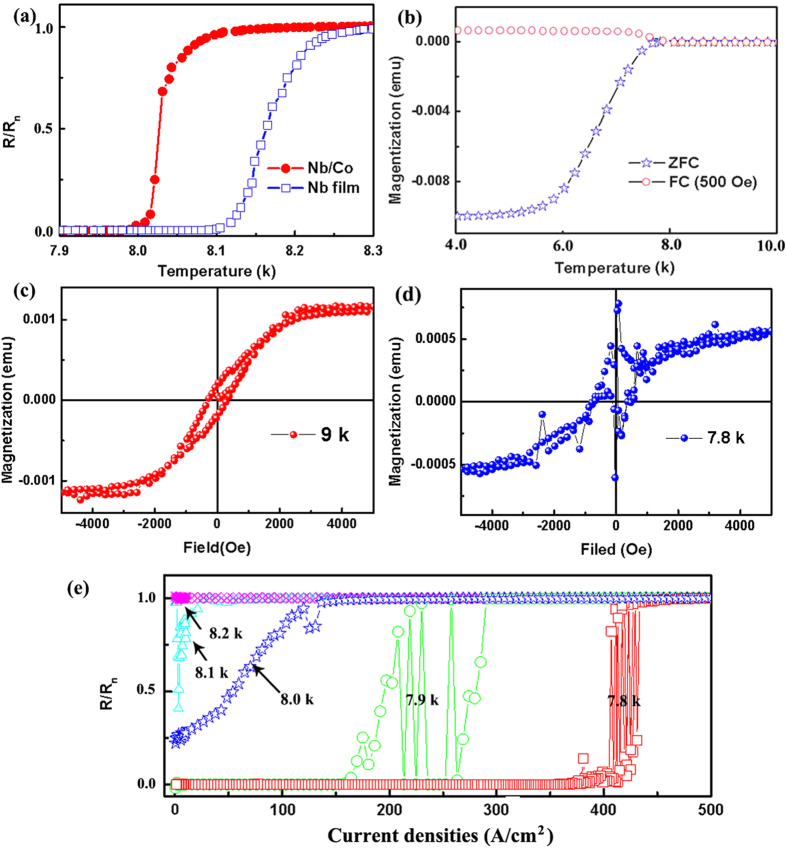
(**a**) Resistance vs temperature curves of Co/Nb hybrid and Nb film; (**b**) ZFC and FC curves of Co/Nb hybrid; (**c**) hysteresis loop of hybrid at 9.0 K, hybrid film is in NS; (**d**) hysteresis loop of hybrid at 7.8 K, hybrid film is in SS; (**e**) I–V curves with different temperatures of Co/Nb hybrid.

Figure 2(a) shows the superconducting transition of Co/Nb hybrid and reference sample Nb film with FE-SEM image in supplementary Fig. S2. Here *R*_*n*_ is resistance of normal state. It can be seen from Fig. 2(a) that the superconducting transition temperature (T_C_) of Co/Nb is lower than that of pure Nb film. It is worth noting that the rate of resistance change in the superconducting transition region between Co/Nb and pure Nb film is different. It means that the superconductivity of Nb is modulated by the Co nanostructured array. Field cooling (FC) and zero field cooling (ZFC) magnetization measurements under an applied field of 500 ○e were shown in Fig. 2(b). When the temperature is below 8 K, ZFC curve displays diamagnetic behavior due to the diamagnetism of superconductor, while FC curve displays paramagnetic Meissner effect^40^.

The following hysteresis loops of Co/Nb hybrid reveal a special magnetic change around *T*_*C*_. Above *T*_*C*_, for example 9.0 K, the hysteresis loop displays a typical ferromagnetic behavior, as shown in Fig. 2(c). Below *T*_*C*_, for example 7.8 K, the hysteresis loop reveals that superconductivity and ferromagnetism co-exist in Co/Nb hybrid, see Fig. 2(d). The hysteresis loops of the pure Nb film at different temperatures are shown in supplementary Fig. S3. The absence of ferromagnetic behavior in Nb film proves ferromagnetism in hybrid comes from Co shell array.

Figure 2(e) shows the I-V curves of hybrid film at different temperatures around *T*_*C*_. At 7.9 K, when the current density exceeds 158 A/cm^2^, the resistance increases from zero to *R*_*n*_ gradually. After the resistance almost approaching *R*_*n*_, the resistance begins to oscillate between zero and *R*_*n*_. When the current density increases beyond 263 A/cm^2^, the oscillation disappears with resistance increasing monotonically from zero to *R*_*n*_. With increasing current, the hybrid film experiences superconducting to normal state transition twice, hence, we term the double-transition.

At 7.8 K, the current corresponding to the oscillation region is greater than 410 A/cm^2^. However, the double-transition is replaced by binary-oscillation-transition in the superconducting transition region. Correspondingly, the I-V curves of Nb film with different temperature are shown in supplementary Fig. S4. For Nb film, the special transition in hybrid is not observed. It indicates that such behavior should be associated with the typical F/S hybrid structure.

**Figure 3:**
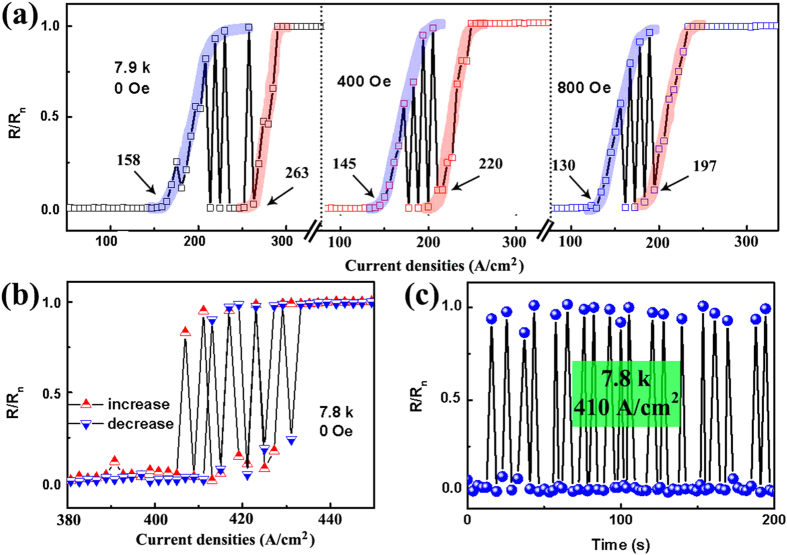
(**a**) I–V curves at 7.9 K, corresponding to double-transition, with different magnetic fields: 0, 400 Oe and 800 Oe. (**b**) I-V curves at 7.8 K, corresponding to oscillation-transition, the red and blue triangles stand for current densities increasing (0–500 A/cm^2^) and decreasing (500-0 A/cm^2^), respectively. (**c**) resistance vs time at 7.8 K with a current density within oscillation region.

Further analysis of the binary transition behavior is displayed in Fig. 3. Figure 3(a) shows I-V curves under different fields at 7.9 K. Double-transition could be observed in both of them. Each transition curve has similar tendency, which indicating the two transitions are correlated. With increasing field, the current corresponding to the oscillation region decreases, and oscillation region is narrowed down as well. This indicated the magnetic field could adjust double-transition.

Figure 3(b) displays the influence of current densities increasing (0-500 A/cm^2^) and decreasing (500-0 A/cm^2^) on the oscillation at 7.8 K. Both of them exists the binary-oscillation-transition, and the two oscillation regions match with each other. To better understand the binary-oscillation-transition, a typical resistance vs time curve in the oscillation region is shown in Fig. 3(c) with a current density of 410 A/cm^2^. It can be seen from Fig. 3(c) that the resistance is only oscillating between SS and NS, like binary code “0” and “1”. This will provide a potential application for low-energy electronic devices, especially smart devices, through controlling the “0” and “1”^41^.

## Discussion

**Figure 4:**
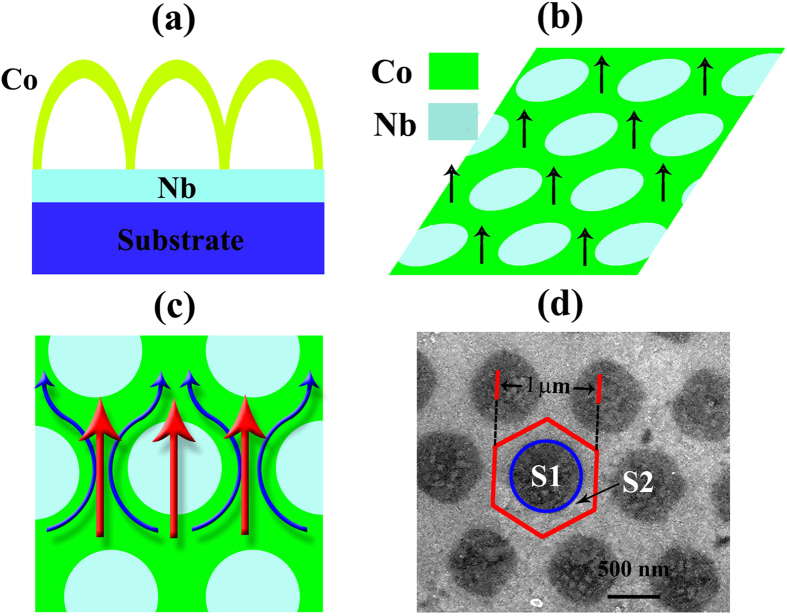
Illustration of the superconductor transition. (**a**) Side view of Co/Nb hybrid film, (**b**) interface between Co and Nb, (**c**) current pass through the Co/Nb film, the red arrows show current pass through evently, while blue arrows show the current through superconductor channel when the core in NS. (**d**) FE-SEM image of interface, here *S*_*1*_ and *S*_*2*_ corresponding to the average area of core and frame, respectively.

Such unusual behaviors should be associated with the typical Co/Nb hybrid structure. In our case, Co hexagon shell array lays on the top of Nb film, as illustrated in Fig. 4(a). At the bottom of shell array, Co contacts the Nb film with a honeycomb pattern thus creating a honeycomb magnetic template to modulate the superconductivity of Nb film, as shown in Fig. 4(b). Because of magnetic pinning as well as proximity effect, the critical current density of Nb film in the area of honeycomb frame (*J*_*FC*_) should be different from that in the area of cores (*J*_*CC*_)^18^,^42^,^43^,^44^,^45^,^46^. If the critical current density at honeycomb frame is higher, with increasing current *I*, the superconductivity in the cores area will be suppressed first, which causes redistribution of the superconducting current from uniformly flowing through the film into being guided along the honeycomb frame. On the contrary, if the critical current density at cores area is higher, the superconductivity will be confined in the cores which forms ordered superconducting islands when the frame just reaches NS. Both superconducting islands and superconducting channel along frame could lead to unusual superconductivity, however, only the former provided a possible way to make resistance oscillate between NS and zero as discussed below, which demonstrates that the frame has higher critical current density.

At low current, both core and frame are in SS, the superconducting current flows uniformly though the film which defines an effective current density *J*, as illustrated by the red arrows in Fig. 4(c). With increasing *J* above *J*_*CC*_, the superconducting current is confined along the honeycomb frame (blue arrows shown in Fig. 4(c)), thereby the effective current density suffered by the frame is enhanced from *J* to *J*_*F*_. If *J*_*F*_ is lower than *J*_*FC*_, zero resistance can be still observed. When *J*_*F*_ reaches *J*_*FC*_, the superconductivity in the honeycomb frame will be also destroyed subsequently. Hence, the first resistive transition is observed. Then, both the frame and core are in NS and the current is restored to uniformly flowing through the film. However, the flow in the whole film will cause the effective current density decreasing from *J*_*F*_ to *J* again. Since *J* is still lower than *J*_*FC*_, the frame will return to SS immediately. Accompanied by the conducting channel changing between the whole film and honeycomb frame back and forth, the effective current density oscillates between *J* and *J*_*F*_. This is the reason that the resistance begins to oscillate between *R*_*n*_ and zero. When *J* is further increased above *J*_*FC*,_ the oscillation ceases and the second resistive transition takes place, see Fig. 3(a).

To confirm the above argument, we can check the ratio of current density corresponding to the two resistive transitions. The first transition of resistance from SS to NS is due to *J*_*F*_ reaching *J*_*FC*_ (current along honeycomb frame), while the second is due to *J* reaching *J*_*FC*_ (current uniformly flowing through the film). Hence, the current density obeys the following formula:


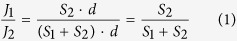


here, *d* is the thickness of Nb film, *J*_*1*_ and *J*_*2*_ are initial points corresponding to first and second transition in I-V curves, S_1_ and S_2_ are the areas of core and frame respectively as shown in Fig. 4(d). In our case, the average diameter of core is about 650 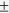 30 nm. The ratio of *S*_*1*_*/S*_*2 *_ can be calculated to be:


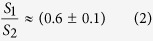


Taking the initial points in Fig. 3(a)–(c) into Equation(1), we obtain the ratio of *S*_*1*_*/S*_*2*_ to be 0.66, 0.55 and 0.52 corresponding to 0, 400 Oe and 800 Oe, respectively. All those ratios are within the error range of Equation(2).

In the above argument, we assure that the first transition of resistance from SS to NS is due to *J*_*F*_ reaching *J*_*FC*_. However, with increasing *J* above *J*_*CC*_, after the superconducting current being confined along the honeycomb frame, it is possible that the effective current density suffered by the frame *J*_*F*_ is higher than *J*_*FC*_. Then the superconductivity in the honeycomb frame will be destroyed immediately and successive resistive transition could not be observed, this is just the case at 7.8 K. To confirm the above hypothesis, R-T and I-V curves of a reference sample with higher area ratio of core to frame is shown in supplementary Fig. S5. Figure S5 (a) shows the superconducting transition with a midpoint at 8.04 K, which almost with the same value of the hybrid sample (8.03 K) shown in Fig. 2(a). Reference sample at 7.9 K shows a binary-oscillation-transition in I-V curve, see Fig. S5 (b), which is different with that of hybrid sample at 7.9 K, but with similar tendency to that at 7.8 K, see in Fig. 2(b). In such shell/film hybrid, different morphologies of contact area mean that the ratio of core/frame is different. Obviously, there exist two possibilities depending on the morphologies of contact area. For lower ratio of core/frame, both double-transition and binary-oscillation-transition states can be observed. However, for higher ratio of core/frame, only the binary-oscillation-transition displays once the superconducitivity in the core areas is destroyed with increasing current. Therefore it provides a simple way to modulate critical current densities of such hybrid by tuning the ratio of core/frame, or different morphologies of contact area.

In summary, a large scale Co/Nb hybrid has been fabricated via colloidal lithography, and Co hexagon shell array modulated critical current densities of Nb film in the hybrid have been performed. An unusual double-transition corresponding to different critical current densities was first observed in I-V curves. By adjusting temperature and contact area of F/S, the double-transition could be replaced by a binary-oscillation-transition. Such novel behaviors attribute to the special magnetic template modulating superconductivity in F/S hybrid. Our investigations have important implications for understanding the role of magnetic subsystem modulating superconductivity in designing superconductor devices for low-energy electric technologies.

## Methods

### Materials

Polystyrene (l μm) suspensions were bought from Duke Corporation. Nb (99.99%) and Co (99.99%) targets were purchased from Kejing Materials Technology Co. Alcohol (99.7%) was obtained from Zhejiang Chemicals. All chemicals were used as received without further purification. Glass and silicon substrates were cleaned according to previously published procedures^30^,^31^.

### Preparation of Nb film

Nb thin films were prepared from Nb target on a silicon substrate by DC magnetron sputtering (DE 500). The base pressure of the sputtering chamber was better than 10^−5 ^Pa. Pre-sputtering and sputtering time is 3000 s and 5000 s, respectively. The sputtering power is 50 W and deposition rate is ~0.03 nm/s. The reference sample Nb film is fabricated with same condition. The reference Nb film is fabricated in the same condition.

### Preparation of Co/Nb hybrid film

A large area and high ordered colloidal monolayer was synthesized via self-assembly at the air-water interface^47^, see supplementary Fig. S6. A prepared silicon substrate with Nb film was inserted beneath the colloidal crystal monolayer, and then lifted the film from the water surface, followed by heating to 60 °C in an oven for 30 min to fix the monolayer with substrate. Subsequently, the samples were put into DC magnetron sputtering (DE 500) to grow a 70 nm thick Co layer on the colloidal template. Finally, the large area hybrid films were formed by baking at 300 ^o^C in vacuum to remove PSs.

### Characterization

The morphology of as-prepared samples was observed by field-emission scanning electron microscopy (FE-SEM) (Hitachi S-4800). The transport properties of samples were measured on a physical property measurement system (PPMS). The magnetic properties of samples were characterized by the superconducting quantum interference device (SQUID) magnetometer.

## Additional Information

**How to cite this article**: Li, Z. *et al.* Magnetically modulated critical current densities of Co/Nb hybrid. *Sci. Rep.* **5**, 18601; doi: 10.1038/srep18601 (2015).

## Supplementary Material

Supplementary Information
