# Supplementary material for: Magnetically modulated critical current densities of Co/Nb hybrid
Source: Sci Rep. 2015 Dec 18;5:18601. doi: 10.1038/srep18601 (PMC4683466; doi:10.1038/srep18601)
Supplement: Supplementary Information [file srep18601-s1.pdf]

Supplementary files

Magnetically modulated critical current densities of Co/Nb hybrid

Zhigang Li[1, 2], Weike Wang[1, 3], Li Zhang[2], Zhaorong Yang[1, 3,4], Mingliang Tian[1,4] & Yuheng Zhang[1,4]

[1]High magnetic field laboratory, Chinese Academy of Sciences, Hefei 230031, P. R. China

[2]Department of Physics & Electronic Engineering, Taizhou University, Taizhou 318000, China.

[3]Key Laboratory of Materials Physics, Institute of Solid State Physics, Chinese Academy of Sciences, Hefei 230031, China

[4]Collaborative Innovation Center of Advanced Microstructures, Nanjing University, Nanjing

210093, China.

*Author for correspondence: Zhaorong Yang Email: zryang@issp.ac.cn

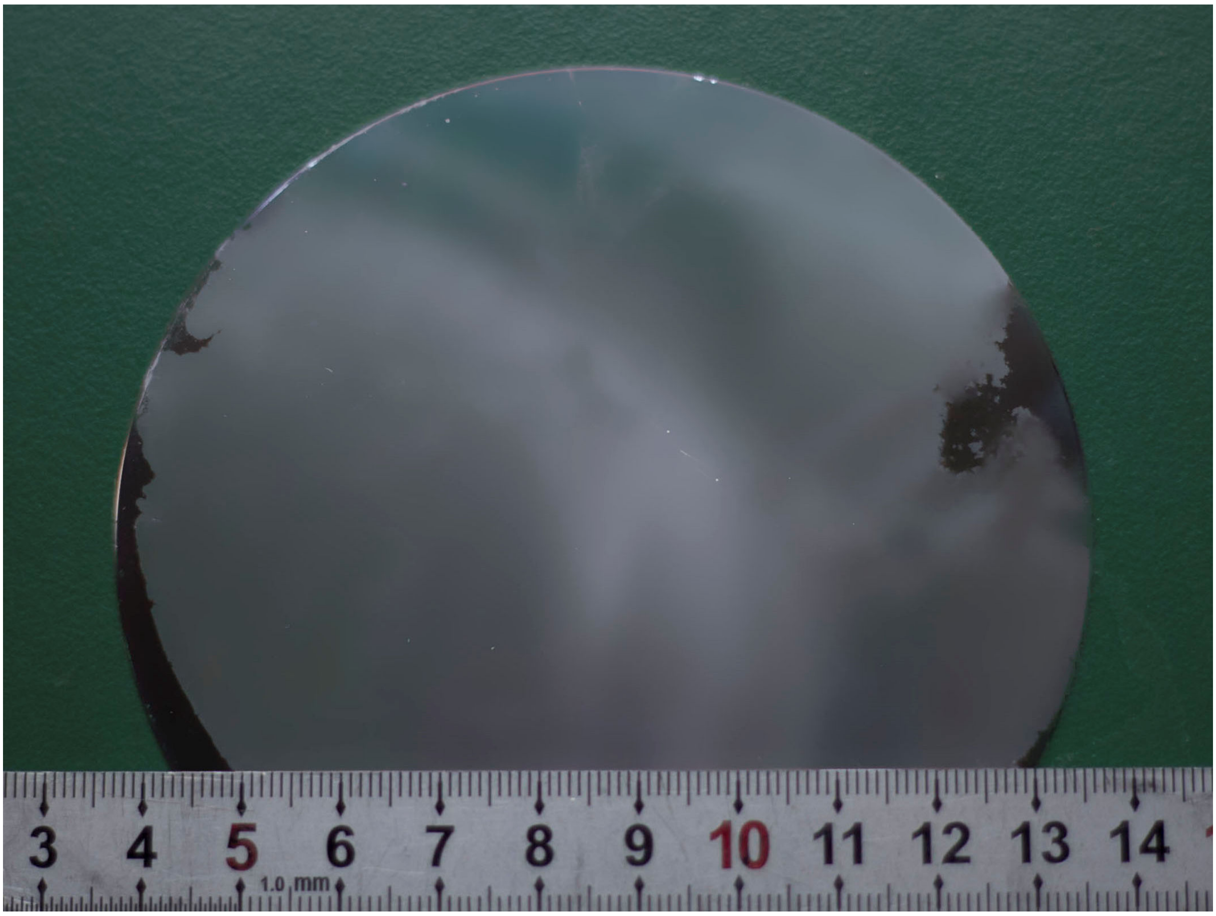

Figure S1. Photography of PSs template on silicon substrate with large area about 80 cm^2.

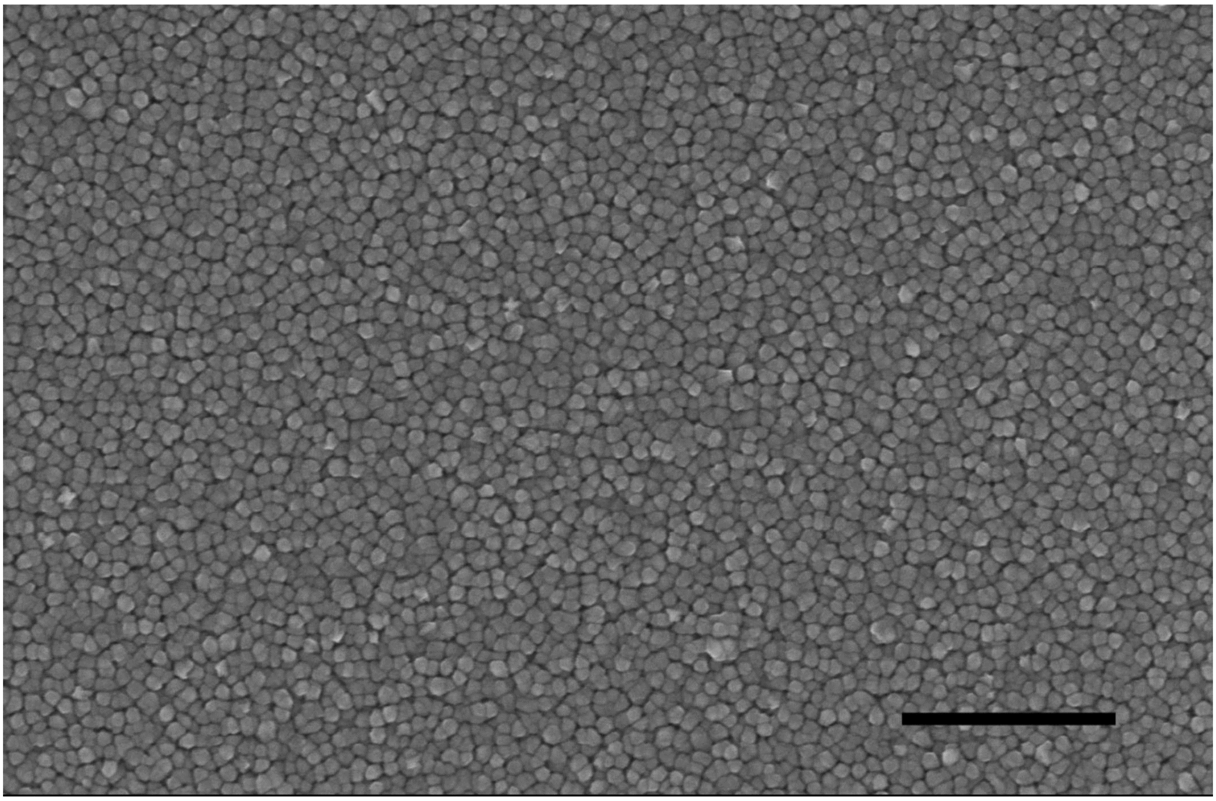

Figure S2. SEM image of Nb film with particle size about 40 nm, the scale bar is 500 nm.

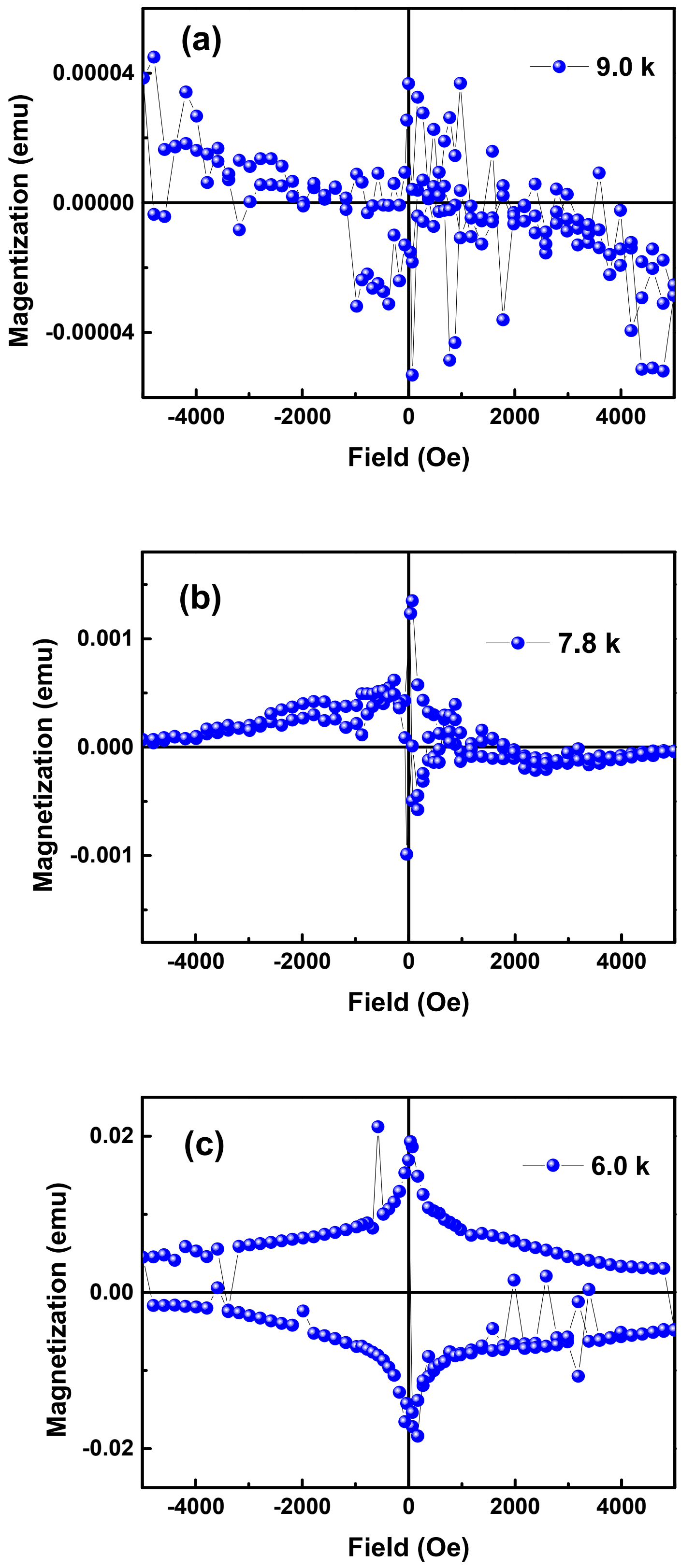

Figure S3. Nb film M-H curves with different temperature. (a) 9.0 K, (b) 7.8 K, (c) 6.0 K.

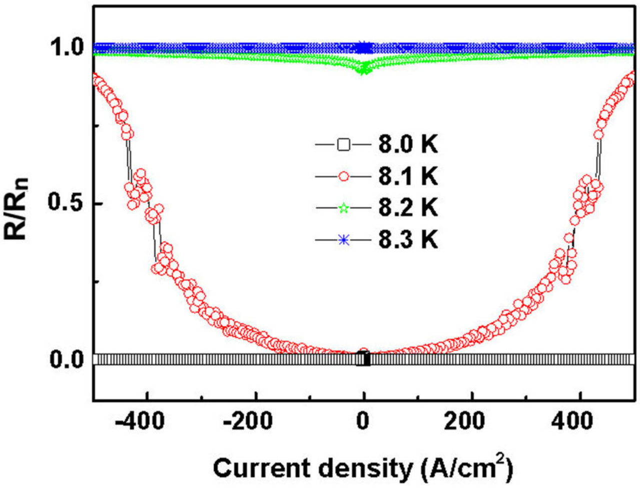

Figure S4. I-V curves of Nb film with different temperatures.

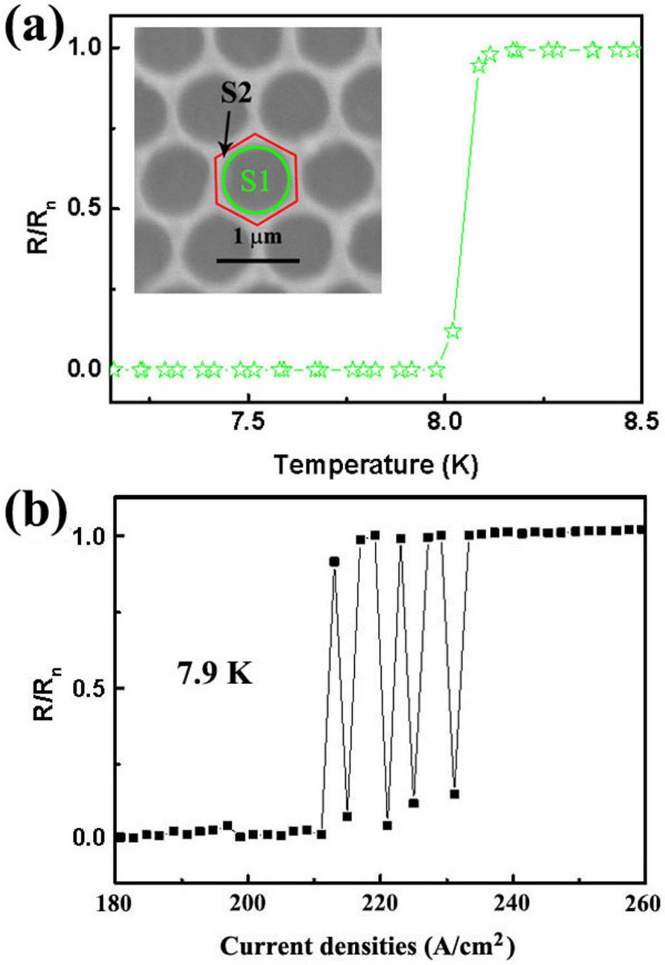

Figure S5. (a) R-T curve of reference hybrid sample, the inset is SEM image. Here, S1 and S2 corresponding to the average area of core and frame, respectively. (b) I-V curve of reference sample at 7.9 K (0.98 Tc).

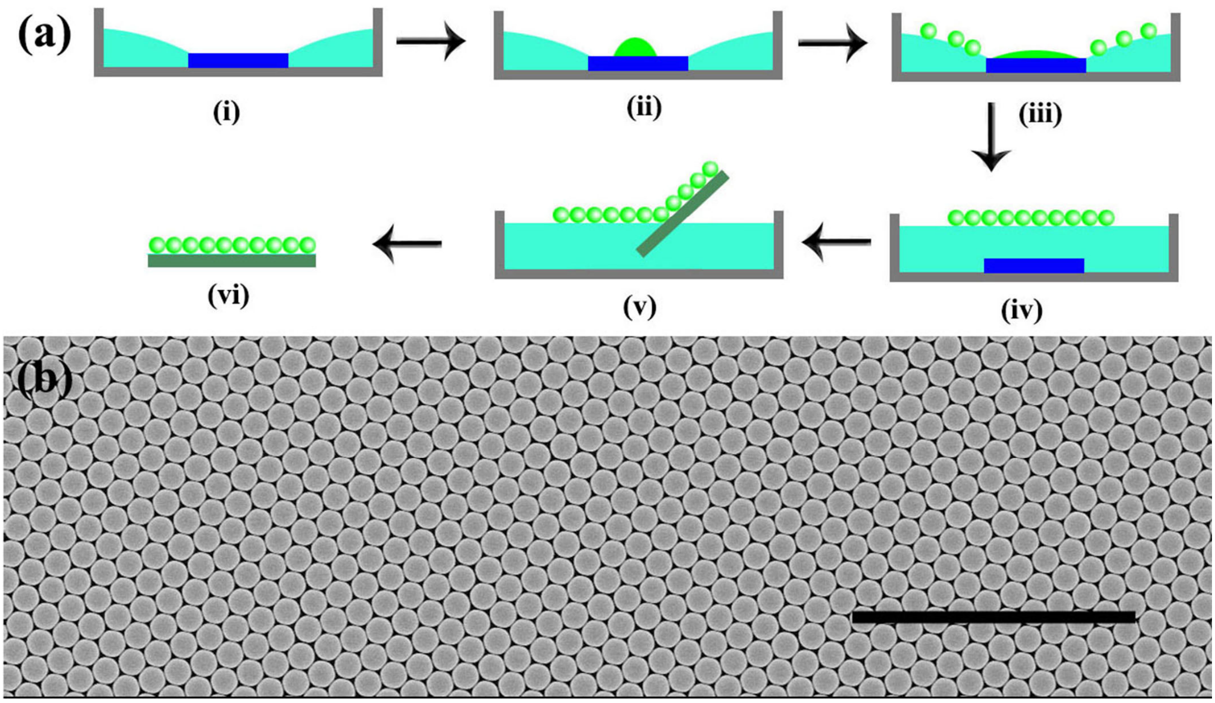

Figure S6. (a) Schematic diagram for the fabrication of PS array: (i) Some pieces of ordinary glass substrates were first ultrasonically cleaned in turn with acetone, ethanol, 3:1 98%H_2SO_4/H_2O_2, 5:1:1 $H_2O/NH_3H_2O/H_2O_2$ and distilled water for 1 h, respectively. A cleaned glass slide was placed in the center of a Petri dish. Deionized (DI) water was then carefully added into the dish to a level where a convex shape was formed around the periphery of the glass slide but without covering the upper surface of the slide. (ii) Five microliters of 10 wt% monodisperse polystyrene colloidal sphere with diameter of 1μm (purchased from Duke Corporation) mixed with ten microliters of DI water and ten microliters of ethanol. Then the mixed colloidal suspension was dropped and spread freely on the glass slide. (iii) Once the suspension contacted the surrounding DI water at the edges of glass slide, it was observed that the PS spheres spread on the water surface rapidly, (iv) assembled into 2D arrays in several seconds at the air-water interface. (v) A required substrate was inserted beneath the colloidal crystal monolayer and then used to lift the film from the water surface as a whole, (vi) then colloidal template will be obtained by drying at room temperature. (b) SEM image of PSs template with diameter 1 μm, the scale bar is 10 μm.
